# Children's Country Holidays Fund

**Published:** 1918-08-03

**Authors:** A. F. London


### THE EDITOR'S LETTER-BOX.

### Children's Country Holidays Fund.

**Who Has the Heart Not to Give Something?**

SIR,—Many of your readers may have noticed at the stations recently parties of children travelling to the country under the auspices of the Children's Country Holidays Fund, all full of excitement and eager anticipation of the joys of the fortnight to come.

At the beginning of August other children will be sent, and the Fund's visitors are besieged with applications from parents anxious to secure a fortnight's change for flagging children, many of whom have been victims of influenza. Subscriptions to the Fund have fallen very considerably since the war, and are still falling. On the other hand, the expenses in every direction have increased, and additional donations are required for this year's work.

Cheques made payable to the Fund should be sent to the Secretary, C.C.H.F., 18 Buckingham Street, Strand, W.C. 2.—Yours faithfully,

(Signed) A. F. LONDON.

